# In Vivo Evaluation of the Anti-Skin-Ageing Bioactivity of a Recombinant Dual Humanised Collagen and Poly-L-Lactic Acid

**DOI:** 10.3390/bioengineering12050510

**Published:** 2025-05-12

**Authors:** Mingjie Tong, Xin Zhou, Jiongni Zhong, Dengjian Qu, Wei Chen, Chun Chen, Yiting Wang, Yaoping Liu, Shaochuan Li, Yuan Xiao, Ning Wang, Chaowan Guo, Qiuling Xie, Sheng Xiong

**Affiliations:** 1 Institute of Biomedicine and National Engineering Research Center of Genetic Medicine, College of Life Science and Technology, Jinan University, Guangzhou 510632, China; tongmingjie0213@163.com (M.T.); xinzhou@stu2021.jnu.edu.cn (X.Z.); z15297776952@126.com (J.Z.); qudengjian@163.com (D.Q.); chenchun_jnu@163.com (C.C.); wyt2010@126.com (Y.W.); txql@jnu.edu.cn (Q.X.); 2 Guangzhou Huike Biotech Co., Ltd., Guangzhou 510530, China; weichen_ray@163.com; 3 Guangzhou Cheer-Derm Biotech Co., Ltd., Guangzhou 510530, China; 18520645363@163.com; 4 College of Veterinary Medicine, South China Agricultural University, Guangzhou 510642, China; shaochuan@scau.edu.cn; 5 Guangdong Marubi Biotech Co., Ltd., Guangzhou 510530, China; xiao.yuan@marubi.cn (Y.X.); wang.ning@marubi.cn (N.W.); guo.chaowan@marubi.cn (C.G.)

**Keywords:** recombinant collagen, skin ageing, TGF-β signalling, PLLA, injectable biomaterials

## Abstract

This study introduces a novel recombinant humanised collagen (DuCol) developed through codon optimisation and prokaryotic soluble expression, exhibiting exceptional biocompatibility and bioactivity. Structural integrity was confirmed via RP-HPLC, SEM, and CD spectroscopy. In vitro evaluations revealed DuCol’s dose-dependent enhancement of NIH-3T3 fibroblast proliferation, adhesion, and migration. In a D-galactose-induced ageing rat model, subcutaneous implantation of DuCol showcased time-dependent anti-ageing effects. Early-stage intervention (30 days post-injection) markedly upregulated COL1A1 expression through the TGF-β/Smad3 pathway activation, outperforming poly-l-lactic acid (PLLA) in collagen deposition. Histological analysis revealed 23.4% greater dermal thickness in DuCol-treated groups compared to PLLA at 90 days. While PLLA exhibited sustained collagen stimulation beyond 90 days, DuCol exhibited superior early-phase efficacy (*p* < 0.001) with comparable safety profiles (no inflammatory response observed through 180-day monitoring). The combinatorial PLLA/DuCol (P&C) formulation synergistically enhanced dermal regeneration, achieving a 31.7% thicker collagen matrix than monotherapy groups. These results underscore the potential of DuCol as a novel implantable filler material for skin repair and regeneration.

## 1. Introduction

Collagen, a fibrillar structural protein constituting approximately 30% of total vertebrate protein content, serves as a vital naturally-derived biomaterial due to its exceptional biocompatibility and hierarchical organisation [1]. This evolutionarily conserved extracellular matrix component demonstrates unique mechanical-elastic properties, enzymatic degradability, and cellular interactivity that underpin its clinical utility in regenerative medicine, tissue engineering (particularly Type I/III collagens), and advanced therapeutic applications [2]. Conventional collagen isolation employing acidic and alkaline extraction protocols from xenogeneic sources typically yields macromolecular assemblies approaching 300 kDa—a benchmark reflecting intact triple-helical integrity [3]. However, this native supramolecular configuration frequently manifests poor bioavailability in clinical applications due to its inefficient cellular internalisation of high-molecular-weight aggregates. Crucially, the harsh chemical milieu (including hypertonic saline buffers and prolonged low-pH exposure) induces deleterious conformational modifications, particularly in denaturation-prone telopeptide regions, causing measurable impairment of collagen’s bioactive motifs [4,5]. Therefore, short collagen peptides with smaller molecular weights and simpler structures obtained by enzymatic hydrolysis are widely used in the beauty and skin care industry. Researchers have demonstrated that short collagen peptides enhance fibroblast proliferation and migration through upregulation of hyaluronan synthase 2 (Has2) expression, a process potentially mediated by the signal transducer and activator of the transcription 3 (STAT3) signalling pathways. This molecular mechanism consequently stimulates collagen and hyaluronic acid biosynthesis, thereby attenuating cutaneous senescence. Emerging evidence suggests that orally administered hydrolysed collagen peptides may exert bioactive effects on skin physiology. Specific dipeptides (e.g., prolyl-hydroxyproline) resist gastrointestinal degradation through PEPT1-mediated absorption and act as signalling molecules to stimulate dermal fibroblasts via chemokine receptors (CXCR4), thereby enhancing collagen synthesis and extracellular matrix remodelling [6,7]. Clinical trials have demonstrated these effects in statistically robust designs: a double-blind, placebo-controlled study (*n* = 106) reported a 15.8% increase in dermal collagen density (*p* < 0.01) and a 19% reduction in transepidermal water loss (TEWL) after 8 weeks of supplementation [8]. Similarly, another randomised trial (*n* = 69) showed significant improvements in skin elasticity (+7.5%, *p* = 0.03) and hydration [9], supporting the potential of oral collagen peptides in mitigating age-related skin changes.

Subcutaneous collagen injections are now established treatments in medical aesthetics and cosmetic surgery. The ideal material for facial tissue fillers must be safe, effective, and biocompatible. Owing to these inherent advantages, collagen’s clinical efficacy in microsurgical plastic procedures is widely recognised. It is primarily used to correct congenital soft tissue defects, diminish tear troughs and nasolabial folds, eliminate postoperative scars, and model human tissue contours [10,11]. Contemporary aesthetic medicine predominantly utilises xenogeneic collagen sourced from animal derivatives, as exemplified by FDA-approved Zyderm^®^—a standardised bovine-derived injectable filler containing 95% type I and 5% type III collagen fractions. This cross-species biomaterial is specifically engineered for deep dermal implantation targeting rhytid correction and volumetric restoration of facial contour depressions, demonstrating a therapeutic window of 3–6 months post-administration [12,13]. Current biomedical practice reveals that more than 98% of collagen employed in therapeutic formulations and tissue-engineered medical devices originates from animal sources, with bovine-derived variants maintaining priority in clinical applications due to established quality-controlled production pipelines and superior batch-to-batch reproducibility [14,15]. Nevertheless, xenogeneic collagen applications present significant clinical challenges, including zoonotic pathogen transmission risks, immunogenic hypersensitivity responses, and batch-dependent antigenicity profiles [16,17]. For example, bovine spongiform encephalopathy (BSE) is a major limiting factor for the use of bovine collagen, especially materials sourced from BSE-affected regions. Additional constraints include socio-religious acceptability barriers in certain demographics, stringent purification requirements to remove non-collagenous epitopes, and inherent inter-batch heterogeneity in macromolecular assembly. Advances in genetic engineering and synthetic biology now enable the rational design of recombinant collagen with programmable structural motifs, positioning it as a next-generation biomaterial for precision surgical implants and regenerative matrix fabrication [15,18]. Production typically involves cloning human-derived collagen gene sequences into heterologous expression systems (e.g., yeast, bacteria, or animal cells), leveraging their biosynthetic machinery to achieve high-yield production with superior batch-to-batch consistency compared to animal-derived counterparts [19]. Crucially, recombinant platforms preferentially utilise human collagen-encoding genes to replicate native fibrillar architectures, thereby maximising host biocompatibility while eliminating species-specific immunogenic epitopes. Cosmoderm and Cosmoplast are collagen implants based on recombinant humanised collagen, originally designed for the treatment of burns [20]. In contrast to animal-derived collagen implants, these recombinant humanised collagen products obviate the need for pre-treatment skin testing, underscoring their enhanced biological safety profile.

In human dermis, the extracellular matrix contains mainly type I and type III collagen, with type I comprising approximately 70% and type III approximately 10% [21]. Therefore, we designed and synthesised the recombinant humanised I/III chimeric collagen (dual collagen, DuCol), demonstrating its efficacy in *C. elegans*, cells, and topical human facial applications. DuCol’s structural integrity was confirmed via RP-HPLC (93.6% purity), SEM (fibrous porous architecture), and circular dichroism (CD) spectroscopy. To investigate DuCol’s in vivo biological effects and generate translational evidence for commercialisation, we established a rat skin ageing model through 42 days of subcutaneous D-galactose administration. DuCol was implanted according to a predefined schedule, with observation periods of 30 days (short-term), 90 days (mid-term), and 180 days (long-term). Histological and molecular analyses were employed to evaluate DuCol’s impact on aged skin, exploring material–cell interactions and their effects on bioactive factors and signalling molecules. PLLA, a synthetic biodegradable polymer approved by the FDA for aesthetic applications, has emerged as a gold-standard material for long-term dermal rejuvenation. Unlike conventional fillers that achieve immediate volumisation through physical displacement, PLLA mediates its anti-ageing properties via a distinct neocollagenesis mechanism: subcutaneously injected PLLA microparticles act as a scaffold to stimulate fibroblast recruitment and gradual collagen deposition over 3–6 months [22,23]. Clinical investigations have demonstrated its sustained efficacy, with dermal thickness increasing by 54.9% at 12-month follow-up [24]. This established time-dependent profile—characterised by delayed yet sustained collagen deposition—provides an essential framework for evaluating the early-phase bioactivity of novel collagen-based formulations, like DuCol. We therefore selected PLLA as a positive control within this study to enable a direct comparison of DuCol’s therapeutic kinetics and synergistic potential.

## 2. Materials and Methods

### 2.1. Materials

The collagen sequence was verified and deposited in the NCBI GenBank with the accession number PQ496881. The pET-28a vector and *E. coli* (BL21) were purchased from Sangon Biotech Co., Ltd. (Shanghai, China). Ni-NTA agarose was sourced from GE HealthCare (Chicago, IL, USA). Hematoxylin and eosin (H&E) staining and Sirius red staining detection kits were obtained from Beijing Solarbio Science & Technology Co., Ltd. (Beijing, China). This animal study was approved by the Laboratory Animals Welfare and Ethics Committee of Jinan University. Ninety-six 8-week-old male SD rats (Sibeifu Biotechnology Co., Ltd. (Beijing, China) SCXK (Jing) 2019-0010) weighing 200 ± 5 g were used for the in vivo experiments. All the rats were housed in an animal laboratory facility (22–24 °C, 50% humidity) under a 12-h light/dark cycle for 7 days to adapt to the environment before surgery.

### 2.2. Expression and Purification of DuCol

The optimised DuCol sequence was cloned into the pET-28a vector, which was then transformed into *E. coli* BL21. Protein expression was induced by IPTG. The cells were harvested through centrifugation at 10,000× *g* for 10 min. The cell pellet was resuspended in NTA-10 buffer (20 mM Tris-HCl, 150 mM NaCl, and 10 mM imidazole, pH 8.0) at a volume ratio of 1:10. Subsequently, the cells were homogenised under high pressure (800 bar). The supernatant was obtained by centrifugation at 10,000× *g* for 30 min. The supernatant was loaded onto a Ni-NTA affinity chromatography column at a flow rate of 0.6 mL/min, and NTA-10 buffer was used to wash back to the baseline at a flow rate of 1 mL/min. The impurities were washed with NTA-40 buffer (20 mM Tris-HCl, 150 mM NaCl, 40 mM imidazole, pH 8.0), and the target protein was eluted with NTA-200 buffer (20 mM Tris-HCl, 150 mM NaCl, 200 mM imidazole, pH 8.0). The purified DuCol was determined via SDS-PAGE.

### 2.3. Physical Characterisation of DuCol

The purity of DuCol was analysed using reversed-phase high performance liquid chromatography (RP-HPLC) with a Waters e2695 system (Waters, Milford, CT, USA). The column used was an Xbridge^®^ Peptide BEH C18 Column 300A (5 μm, 4.6 × 150 mm, 1/pkg, Waters, Milford, CT, USA), with mobile phases consisting of 0.1% trifluoroacetic acid (TFA) in deionised water (phase A) and 0.1% TFA in acetonitrile (phase B). Detection was performed at a wavelength of 280 nm, with a flow rate of 1 mL/min and a sample injection volume of 100 μL. Scanning electron microscopy (SEM) was used to characterise the morphology of DuCol. Briefly, lyophilised DuCol samples were mounted onto cleaned copper stubs using conductive carbon tape and subjected to gold sputtering before imaging at a working voltage of 20.0 kV. Fourier-transform infrared (FT-IR) spectroscopy was conducted on a Nicolet 6700 spectrometer (Thermo Fisher Scientific, Waltham, MA, USA), recording spectra in the 4000–400 cm^−1^ wavenumber range. The thermal stability of DuCol was tested by thermogravimetric analysis (TGA) using a Mettler-Toledo TGA 1 Thermogravimetric Analyzer (Zurich, Switzerland) under a nitrogen atmosphere with a flow rate of 50 mL min^−1^ and a heating rate of 10 °C min^−1^. The analysis was performed over a temperature scan range of 35–250 °C. DuCol was tested using a UV-vis spectrophotometer (UV-1750, SHIMADZU, Kyoto, Japan) with scan ranges of 190–400 nm. CD tests were performed on a CD spectrometer (JASCO J1500, Tokyo, Japan) to determine the triple helix structure of DuCol in the wavelength range of 190–260 nm.

### 2.4. In Vitro Biocompatibility and Bioactivity Evaluation

The cytotoxicity of DuCol was evaluated in NIH-3T3 fibroblasts using CCK-8 assays. Cells were seeded at a density of 1 × 10^5^ cells/mL in complete cell culture medium (DMEM containing 10% FBS and 1% penicillin–streptomycin) in 96-well plates and incubated for 24 h in a humidified atmosphere containing 5% CO_2_ at 37 °C. Following the initial incubation, the cell culture medium was aspirated, and a series of concentrations of DuCol solutions diluted with DMEM containing 1% FBS were added to the wells for a further 24 h of incubation. To assess cell viability, 10 μL of CCK-8 reagent diluted in 90 μL of DMEM was added to each well. Plates were incubated in the dark at 37 °C for 1 h before measuring the optical density (OD) at 450 nm using a Tecan Infinite F200/M200 multifunctional microplate reader (Tecan, Männedorf, Switzerland). Measurements were performed for blank (pure DMEM), control (DMEM with 1% FBS), and experimental samples.

Cell proliferation was assessed using a CCK-8 assay to further evaluate the biocompatibility of the samples. Fibroblasts were seeded in 96-well plates and treated with DuCol solutions at various concentrations (diluted in DMEM containing 1%FBS). Cell viability was measured after 48 h of incubation. The OD at 450 nm was measured using a Tecan Infinite F200/M200 multifunction microplate reader (Tecan, Männedorf, Switzerland). Measurements were performed for blank (pure DMEM), control (DMEM with 1% FBS), and experimental samples.

Cell viability (%) = [OD (sample) − OD (blank)]/[OD (control) − OD (blank)] × 100%

The cell adhesion properties of DuCol were assessed using a CCK-8 assay. DuCol solutions at various concentrations were first prepared by diluting with phosphate-buffered saline (PBS). A total of 100 μL of each solution were added to individual wells of a 96-well plate and incubated overnight at 4 °C. Following incubation, the protein solution was aspirated, and 100 μL of a cell suspension (1 × 10^5^ cells/mL) were added to each well. Plates were incubated for 3 h at 37 °C to allow cell attachment. Non-adherent cells were then removed by washing three times with PBS. To quantify cell adhesion, 10 μL of CCK-8 reagent (diluted in 90 μL of DMEM) were added to each well, and plates were incubated in the dark at 37 °C for 1 h. The OD at 450 nm was measured using a Tecan Infinite F200/M200 multifunctional microplate reader (Tecan, Männedorf, Switzerland) for blank (pure DMEM), control (DMEM with 1% FBS), and experimental samples.

Relative adhesion ratio (%) = [OD (sample) − OD (blank)]/[OD (control) − OD (blank)] × 100%

To evaluate cell migration, a scratch assay was performed. Fibroblasts at a density of 1 × 10^6^ cells/mL were seeded into each well of 6-well plates and allowed to form a confluent monolayer. A 10 μL pipette tip was used to create a uniform scratch in the cell monolayer at 0 h. The samples were added to the wells, and the plates were incubated for 8 and 24 h. Cell migration was observed and imaged using an inverted phase-contrast microscope (Olympus IX53, Tokyo, Japan). The migration area at 8 h and 24 h was measured using Image pro-Plus 6.0 software and calculated by comparison with the initial scratched area at 0 h.

### 2.5. Ageing Model

A total of 96 rats were randomly divided into a normal group and an ageing model group, with 48 rats per group. In the ageing model group (D-galactose group), the rats received daily subcutaneous injections of D-galactose solution (300 mg/kg) into the nape of the neck for 42 days. The normal group (0.9% NaCl group) was injected with an equivalent volume of 0.9% sodium chloride solution based on body weight. Rat weights were recorded daily. Modelling success was determined by measuring superoxide dismutase (SOD) activity and malondialdehyde (MDA) levels in plasma. Briefly, the rats were anaesthetised via an intraperitoneal injection of 3% pentobarbital sodium. Orbital blood sampling was performed using a glass capillary tube, with approximately 200 μL blood collected into heparin–sodium anticoagulant tubes. Samples were gently inverted to mix with heparin, then centrifuged at 3500 rpm for 5 min. The upper plasma layer was aspirated, and SOD activity and MDA levels were assayed using commercial kits.

### 2.6. Subcutaneous Injection of Implants

The 48 rats in the 0.9% NaCl group were randomly partitioned into four subgroups, each comprising 12 rats. These subgroups were designated as the blank group, PLLA group, COL (DuCol) group, and P&C (PLLA and DuCol) group, according to the distinct implants administered. Similarly, the 48 rats in the D-galactose group were divided into four equivalent subgroups: the model group, M-PLLA (model-PLLA) group, M-COL (model-DuCol) group, and M-P&C (model-PLLA and DuCol) group. Comprehensive details of the animals within each group are presented in Table 1. The positive control, PLLA, is a water-insoluble microsphere lyophilised powder. Commercially available PLLA microsphere fillers and collagen fillers typically incorporate sodium carboxymethylcellulose as a co-suspending agent, enabling the filler material to form a homogeneous, injectable gel-like solution. Accordingly, in this study, sodium carboxymethylcellulose was utilised as the suspending aid for injection. Following anaesthesia, the dorsal fur of each rat was shaved to an area of approximately 4 × 6 cm. The prepared injection gel was then subcutaneously administered into the rat dorsum using a sterile disposable syringe, with 0.2 mL injected per site. Each injection site was clearly marked upon completion of the injection procedure.

### 2.7. Histological Observation and Quantitative Analysis

The rats were humanely euthanised via ketamine overdose at designated observation time points, with four animals euthanised per group at each time point. Following euthanasia, the treated skin and overlying subcutaneous tissue encompassing the injection site were rapidly excised and rinsed with precooled saline. After gentle blotting to remove surface moisture, tissues were trimmed into fragments measuring approximately 2.0 mm × 13.0 mm. Specimen tissues were fixed in 4% paraformaldehyde for a minimum of 24 h, then dehydrated, paraffin-embedded, and sectioned into 5-μm-thick slices. Masson’s trichrome staining was employed to distinguish between the collagen fibres and myofibres, and the thicknesses of the epidermis and dermis were quantitatively analysed. Sirius red staining was utilised to differentiate among various collagen types within the skin matrix. For any histological staining-based quantitative analysis, a minimum of five distinct fields of view were randomly selected from each animal. The images were analysed via ImageJ 6.0 to determine the proportions of different collagen types, whereas Image-Pro Plus (version 6.0) was applied to measure the thicknesses of the epidermis and dermis.

### 2.8. Gel Electrophoresis and Western Blot

All the samples were combined with 5× SDS-PAGE loading buffer at a 4:1 volume ratio and heated at 100 °C for 10 min. Sodium dodecyl sulfate–polyacrylamide gel electrophoresis (10% SDS-PAGE) was conducted under reducing conditions. For Western blotting, proteins were electrophoretically transferred from the gel to a 0.45 μm nitrocellulose membrane (Merck, Darmstadt, Germany), which was then blocked with 5% non-fat dry milk. The membrane was incubated overnight at 4 °C with primary antibodies: TGFβ-1 antibody (Wuhan Sanying Biotechnology, Inc., Wuhan, China) and Anti-Collagen I antibody (Abcam, Cambridge, UK). Following three washes with 1× TBST buffer, the membrane was probed with an HRP-conjugated anti-rabbit IgG secondary antibody (Wuhan Sanying Biotechnology, Inc., China) for 1 h at room temperature. After additional TBST washes, a chemiluminescent substrate was applied, and the nitrocellulose membrane was imaged using a gel documentation system.

### 2.9. RNA Isolation and Real-Time Quantitative Polymerase Chain Reaction (RT-qPCR)

Total RNA was extracted from tissue samples using TRIzol reagent (Ambion, Inc., Austin, TX, USA) following the manufacturer’s protocol. The extracted RNA was reverse transcribed into cDNA using an iScript^™^ cDNA Synthesis Kit (Bio-Rad Laboratories, Inc., Hercules, CA, USA), generating templates for RT-qPCR amplification. The primer sequences for the *GAPDH*, *COL1A1*, *TGFβ-1*, *Smad3*, *TIMP1*, and *MMP1* genes were synthesised by Qingke Biotechnology Co., Ltd., (Beijing, China) and are detailed in Table 2. An RT-qPCR was performed via real-time quantitative PCR (Bio-Rad CFX96, Hercules, CA, USA) using a Perfectstart^®^ Green qPCR SuperMix System (TransGen Biotech Co., Ltd., Beijing, China). The formula 2^−ΔΔCT^ method was applied to determine the relative quantification of mRNA, which was normalised to the GAPDH gene mRNA level as an endogenous control. Each sample was analysed in triplicate to ensure reproducibility.

### 2.10. Statistical Analysis

All the data are presented as a mean ± standard deviation (SD) and were obtained from multiple independent synthesis batches. Statistical analyses were conducted using GraphPad Prism 8 software. Intergroup differences were evaluated via Student’s *t*-test for pairwise comparisons and one-way analysis of variance (ANOVA) for multi-group comparisons. A *p*-value < 0.05 was considered statistically significant, with significance levels denoted as follows: (*) for *p* < 0.05, (**) for *p* < 0.01, (***) for *p* < 0.001, and (****) for *p* < 0.0001.

## 3. Results

### 3.1. Preparation and Characterisation of DuCol

**Figure 1:**
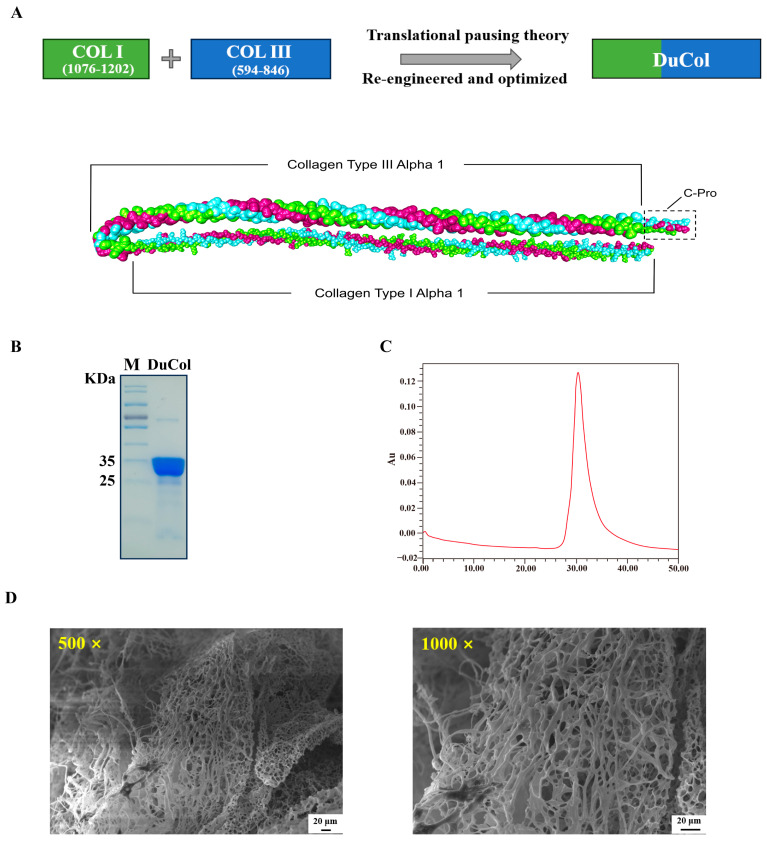
Preparation and characterisation of DuCol. (**A**) Schematic sequence construction of DuCol and the AlphaFold 3-predicted structure of DuCol reveals a trimeric conformation. (**B**). SDS-PAGE analysis of DuCol. The purified protein exhibited to be homogeneous with purity of 94.1%. (**C**) RP-HPLC analysis of DuCol with purity of 93.6%. (**D**) DuCol (SEM 500×; 1000×) exhibited to be typical loose and porous collagen-like structure.

Amino acid residues 1076–1202 of human type I collagen and 594–846 of type III collagen served as templates for designing recombinant humanised collagen. These sequences contain characteristic Gly-X-Y repeat motifs within collagen, incorporating both an integrin-binding structural domain and a pro-cell proliferation domain [25,26]. The nucleotide sequence of DuCol was redesigned and optimised using translation pausing theory [27]. Furthermore, three identical recombinant chimeric collagen monomers were processed through AlphaFold (v3.0) for trimeric structure prediction. Each monomer comprised a chimeric design integrating type I collagen (residues 1–126; COL1A1-derived) and type III collagen (residues 127–261; COL3A1-derived) domains. A decapeptide C-terminal loop was engineered to stabilise triple helix assembly through strategic residue interactions. The computational modelling yielded five putative structures, of which the highest-confidence prediction (pLDDT > 90) is depicted in Figure 1A. A structural analysis revealed a characteristic triple-helical topology demonstrating remarkable congruence with natural collagen architecture. The optimised amino acid sequence of DuCol is shown in Table 3. SDS-PAGE and RP-HPLC were employed to characterise the molecular weight and purity of the expression-optimised DuCol. As shown in Figure 1B, the apparent molecular weight of DuCol was approximately 28 kDa, exceeding the theoretical value of 23.91 kDa. This discrepancy is hypothesised to stem from the high hydrophilicity of the recombinant humanised collagen, which weakens its binding affinity with SDS. Given that SDS-protein interactions predominantly rely on hydrophobic forces, reduced binding results in slower electrophoretic migration and an overestimated molecular weight [28]. Following area normalisation, the purity of DuCol was determined to be 93.6% (Figure 1C). The SEM images revealed that DuCol exhibited a filamentous morphology with many pore-like structures. These structures were more uniform in shape and size compared to other samples (Figure 1D). The FT-IR analysis (Figure S1A) showed that DuCol displayed characteristic collagen absorption peaks. Specifically, amide A and amide B were observed in the hydrogen bonding region (4000–2300 cm^−1^), amide I and amide II in the double-bond stretching vibration region (2000–1500 cm^−1^), and amide III in the fingerprint region (1300–400 cm^−1^). Thermogravimetric analysis is a technique that measures the mass of a sample as a function of temperature, utilising a balance under a controlled, programmed temperature ramp [29]. The thermogravimetric curve represents the cumulative weight loss of the sample during heating. As shown in Figure S1B, the TG curve exhibits two distinct weight loss stages. The first stage occurs below 100 °C, during which the lost water primarily consists of free water and freezable bound water; the second stage initiates above 240 °C, corresponding to the loss of non-freezable water. The UV absorption spectrum represents the collective contribution of various chromophores. Chromophores are fundamentally groups featuring unsaturated π-bonds and lone electron pairs, exemplified by C=C, C=O, and C=S moieties. Aromatic amino acids, such as tyrosine and tryptophan, exhibit characteristic absorption peaks at 280 nm, which arise from π→π* electron transitions [30]. By contrast, DuCol contains minimal aromatic amino acids, resulting in the absence of a 280 nm absorption peak. As shown in Figure S1C, the recombinant collagen displayed an absorption peak at approximately 190 nm. Typically, natural collagen adopts a triple-stranded superhelical architecture, formed by the coiling of three left-handed α-helices. Its CD spectrum is characterised by a prominent negative absorption peak around 198 nm and a smaller positive absorption peak near 220 nm [31,32,33]. During collagen denaturation, however, the 220 nm absorption peak diminishes proportionally to the degree of denaturation, while the 198 nm peak becomes more pronounced. As shown in Figure S1D, the DuCol protein exhibited a distinct negative absorption peak at 198 nm yet lacked a corresponding positive absorption peak at 220 nm.

### 3.2. In Vitro Bioactivity Evaluation of DuCol

**Figure 2:**
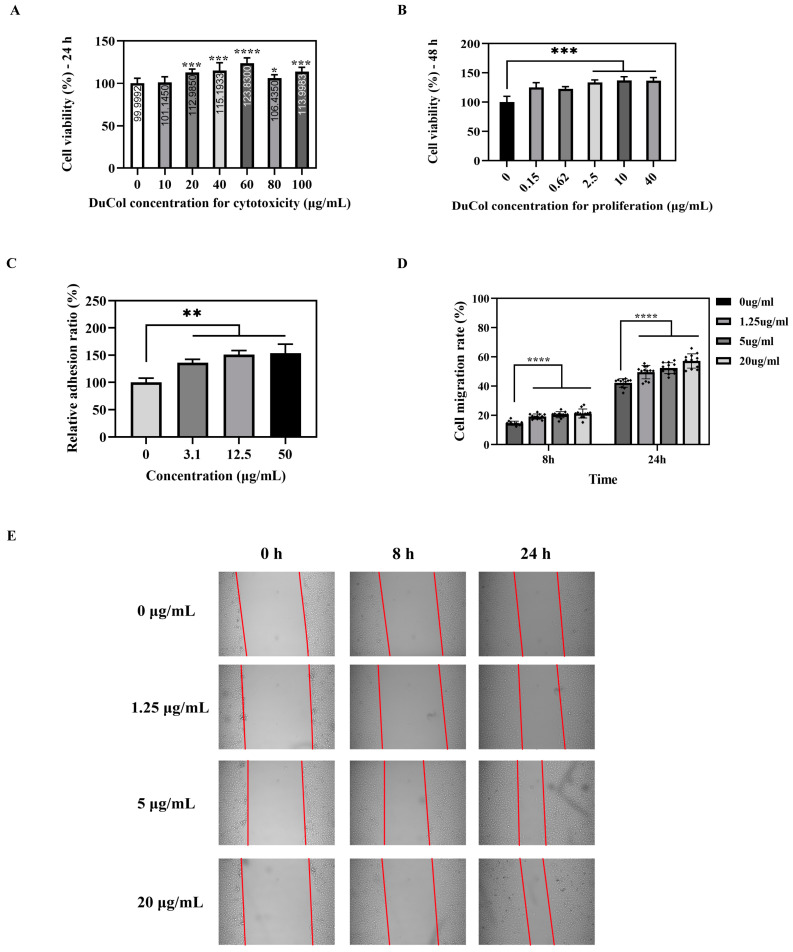
In vitro biocompatibility and bioactivity evaluation of DuCol. (**A**) Cytotoxicity assay of DuCol. NIH-3T3 fibroblasts were treated with increasing concentrations of DuCol (0–100 μg/mL) for 24 h. Cell viability was determined via CCK-8 assay. Data are normalised to untreated controls (DMEM with 1% FBS). No significant cytotoxicity was observed. (**B**) Cell proliferation assay of DuCol. Fibroblasts were incubated with DuCol (0.15–40 μg/mL) for 48 h. (**C**) Cell adhesion assay of DuCol. Wells pre-coated with DuCol (3.1–50 μg/mL) were seeded with 1 × 10^5^ cells/mL for 3 h. Non-adherent cells were removed by PBS washing prior to CCK-8 measurement. (**D**) Statistical graph of cell migration promoted by DuCol. Wound closure area was measured using ImageJ. Data expressed as percentage of initial wound area (0 h). (**E**) Cell migration of DuCol. Representative time-lapse images (0, 8, 24 h) of scratch-wound closure in the presence of DuCol (1.25–20 μg/mL). (*n* = 6; * *p* < 0.05; ** *p* < 0.01; *** *p* < 0.001; **** *p* < 0.0001).

Cytotoxicity assays revealed no significant cell death across all tested concentrations (0.15–100 μg/mL), with cell viability consistently exceeding 100% after 24 h (Figure 2A), confirming DuCol’s biocompatibility and lack of cytotoxic effects. To evaluate proliferative effects, cells were treated with DuCol at 2.5–40 μg/mL for 48 h. As shown in Figure 2B, DuCol exerted a more pronounced proliferative effect across the concentration range of 2.5–40 μg/mL. Cell proliferation rates within this interval were elevated by approximately 30% relative to the control group, with highly significant differences (*p* < 0.001). By comparison, cell adhesion rates on 96-well plates coated with DuCol at 3.1 μg/mL, 12.5 μg/mL, and 50 μg/mL increased by 36.2%, 50.8%, and 53.6%, respectively, compared to the control (Figure 2C), demonstrating DuCol’s excellent pro-adhesive properties in a dose-dependent manner. Cell migration rates were evaluated by calculating the ratio of the wound area difference between 0 h and each time point to the initial wound area at 0 h. As shown in Figure 2D, E, at 8 h, the cell migration rates for the 1.25 μg/mL, 5 μg/mL, and 20 μg/mL doses were 19.1%, 20.4%, and 21.2%, respectively, which were significantly higher than the control group (14.5%, *p* < 0.001). By 24 h, these rates increased to 49.5%, 52.3%, and 57.2%, respectively, in the experimental groups, all notably greater than the control (42.1%, *p* < 0.001). These results demonstrate that DuCol promotes cell migration in a dose-dependent manner with significant efficacy.

### 3.3. Establishment of the D-Galactose-Induced Ageing Model

Excessive D-galactose suppresses the activity of SOD, impairing the timely elimination of free radicals. Additionally, D-galactose generates free radicals during its metabolic oxidation, which trigger lipid peroxidation reactions. This process elevates levels of malondialdehyde MDA, the end-product of lipid peroxidation. Thus, SOD activity and MDA content were measured to validate successful establishment of the ageing model. As shown in Figure S2, compared with the 0.9% NaCl group, the D-galactose group exhibited significantly reduced plasma SOD activity and increased MDA content. These findings indicate that 42 days of continuous D-galactose administration substantially diminished the rats’ antioxidant capacity, confirming successful generation of a skin ageing model.

### 3.4. COL1A1 Expression Level in Skin

**Figure 3:**
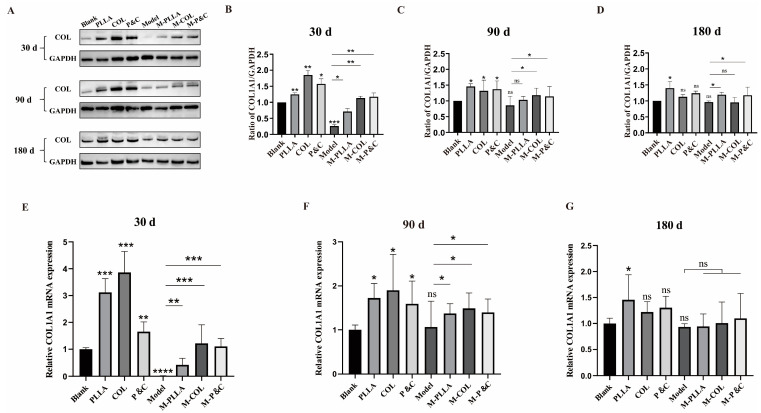
COL1A1 expression levels in rat skin tissues at different time points post-treatment. (**A**) Western blot analysis. Representative immunoblots of COL1A1 and GAPDH (loading control) in skin tissues from the rats treated with DuCol, PLLA, or PLLA/DuCol combination (P&C) at 30, 90, and 180 days. (**B**–**D**) Quantitative COL1A1/GAPDH ratio after 30/90/180 days of injection. Densitometric analysis of Western blot bands normalised to GAPDH. Data expressed as fold change relative to untreated controls. (**E**–**G**) COL1A1 mRNA levels after 30/90/180 days of injection. RT-qPCR analysis of COL1A1 expression normalised to GAPDH. (*n* = 6; * *p* < 0.05; ** *p* < 0.01; *** *p* < 0.001; **** *p* < 0.0001; ‘ns’: *p* > 0.05).

The effects of different implants were evaluated by measuring COL1A1 expression in rat skin following treatment with each implant. The results are shown in Figure 3A–D. At all three time points, dermal COL1A1 expression increased significantly 30 days after injection of filler materials, with the greatest upregulation observed at this early time point compared to 90 and 180 days. This indicated that the three injection materials most potently enhanced COL1A1 expression during the early post-injection period, significantly promoting type I collagen accumulation in skin tissue. While DuCol’s ability to upregulate COL1A1 expression began to diminish by 90 days post-injection, some maintenance effect persisted. By 180 days, PLLA continued to promote COL1A1 expression, whereas DuCol showed no sustained capacity to enhance collagen accumulation—presumably due to subcutaneous degradation of the injected material. Real-time PCR analysis of COL1A1 mRNA levels in skin tissues (Figure 3E–G) corroborated these protein expression findings, demonstrating consistent results across molecular and protein-level assessments.

**Figure 4:**
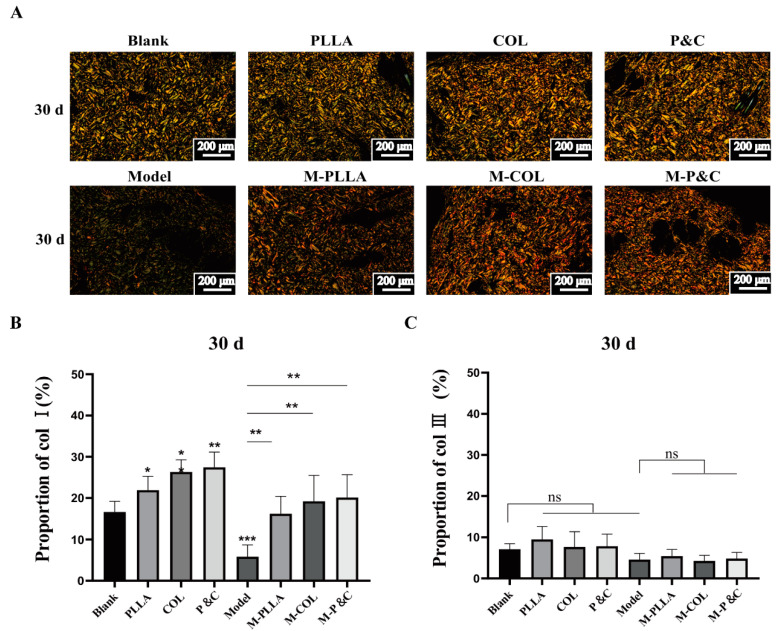
Sirius red staining and collagen content analysis in rat skin tissues 30 days post-injection. (**A**) Representative polarised light microscopy images. Sirius red-stained sections of rat dermis from each treatment group. Type I collagen fibres (red/yellow birefringence) and type III collagen fibres (green birefringence) were differentiated under a polarised light microscope. Scale bar: 200 μm. (**B**) Quantification of type I collagen. Percentage of type I collagen area relative to total dermal area, analysed using ImageJ. (**C**) Quantification of type III collagen. Percentage of type III collagen area relative to total dermal area. No significant differences were observed between treatment groups. (*n* = 6; * *p* < 0.05; ** *p* < 0.01; *** *p* < 0.001; ‘ns’: *p* > 0.05).

Sirius red staining was employed to visualise the distribution and to quantify changes in type I and type III collagen within the dermal layer of skin tissue. Owing to the difference in the refractive index, these collagen types exhibit distinct colours under polarised light microscopy: type I collagen stains red or yellow, while type III collagen appears green [34]. The results are shown in Figure 4. In the early-stage model group, Sirius red-stained sections showed sparser distributions of both red (type I) and green (type III) collagen compared to the blank group, indicating that D-galactose induced dermal damage and loss of both collagen types. After intervention with filler materials, the distribution proportion and density of type I collagen in the dermis increased, whereas type III collagen levels remained unaffected. At 30 days post-injection, dermal type I collagen content rose significantly in all treatment groups, with the COL group demonstrating greater accumulation than the PLLA group. By 90 and 180 days, while all treatment groups retained higher type I collagen percentages compared to the control, the COL group’s efficacy was surpassed by PLLA. The P&C group exhibited higher type I collagen proportions than both the COL and PLLA monotherapy groups. Overall, quantification of dermal type I collagen revealed that DuCol implants outperformed PLLA in the early treatment phase, highlighting DuCol’s superior early-stage capacity to improve skin ageing parameters (Figures S3 and S4). The COL1A1 accumulation in aged skin peaked at 30 days after DuCol injection. Conversely, PLLA demonstrated more favourable pro-collagen expression effects at 90 and 180 days, indicating divergent temporal efficacy profiles between the materials.

### 3.5. TGFβ-1 Expression Level in Skin

**Figure 5:**
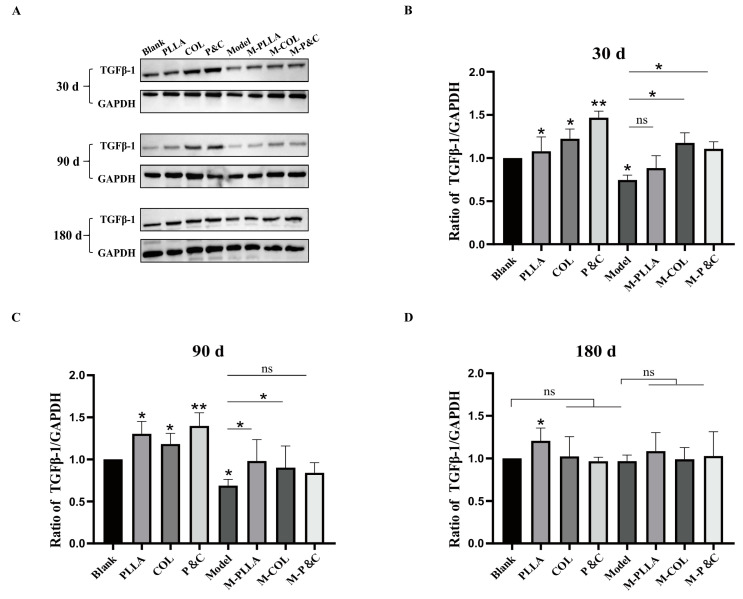
TGFβ-1 expression levels in rat skin tissues at different time points post-treatment. (**A**) Western blot analysis. Representative immunoblots of TGFβ-1 and GAPDH (loading control) in skin tissues from the rats treated with DuCol, PLLA, or PLLA/DuCol combination (P&C) at 30, 90, and 180 days. (**B**) Quantitative TGFβ-1/GAPDH ratio after 30 days of injection. Densitometric analysis of Western blot bands normalised to GAPDH. Data expressed as fold change relative to untreated controls. (**C**) Quantitative TGFβ-1/GAPDH ratio after 90 days of injection. Densitometric analysis of Western blot bands normalised to GAPDH. Data expressed as fold change relative to untreated controls. (**D**) Quantitative TGFβ-1/GAPDH ratio after 180 days of injection. Densitometric analysis of Western blot bands normalised to GAPDH. Data expressed as fold change relative to untreated controls. (*n* = 6; * *p* < 0.05; ** *p* < 0.01; ‘ns’: *p* > 0.05).

**Figure 6:**
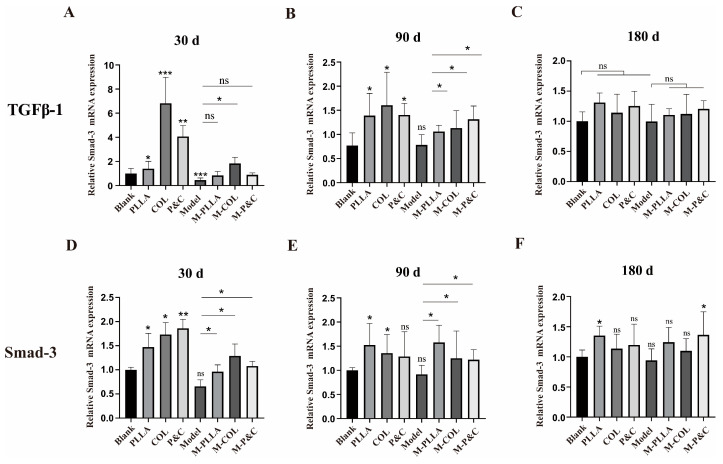
The mRNA expression levels of TGF-β1 and Smad3 in rat skin tissues at different time points post-treatment. (**A**–**C**) TGF-β1 mRNA expression. RT-qPCR analysis of TGF-β1 transcript levels normalised to GAPDH after 30/90/180 days of injection. (**D**–**F**) Smad3 mRNA expression. RT-qPCR analysis of Smad3 transcript levels normalised to GAPDH after 30/90/180 days of injection. (*n* = 6; * *p* < 0.05; ** *p* < 0.01; *** *p* < 0.001; ‘ns’: *p* > 0.05).

To investigate whether the upregulation of COL1A1 in the skin of each experimental group following material intervention correlated with activation of the TGFβ signalling pathway, TGFβ-1 expression levels were assessed in rat skin tissues. The TGFβ/Smad pathway is critical for regulating collagen expression [35]. Upon binding to cell membrane receptors, TGFβ-1 activates the type I receptor (TβRI) within the receptor complex, initiating phosphorylation of downstream Smad proteins—predominantly Smad2 and Smad3. Phosphorylated Smads form a complex with Smad4, which translocates to the nucleus and binds to the collagen gene promoter regions, thereby modulating collagen transcription and expression [36]. As shown in Figure 5, both the COL and M-COL groups exhibited marked increases in TGFβ-1 expression at the 30-day time point, coinciding with the peak COL1A1 expression observed earlier. Figure 6 shows the expression level of Smad3, a key downstream mediator of TGFβ-1 signalling, which mirrored the temporal dynamics of TGFβ-1. These results suggest that filler materials regulate collagen synthesis by engaging the TGFβ/Smad signalling cascade.

### 3.6. Collagen Degradation In Vivo

**Figure 7:**
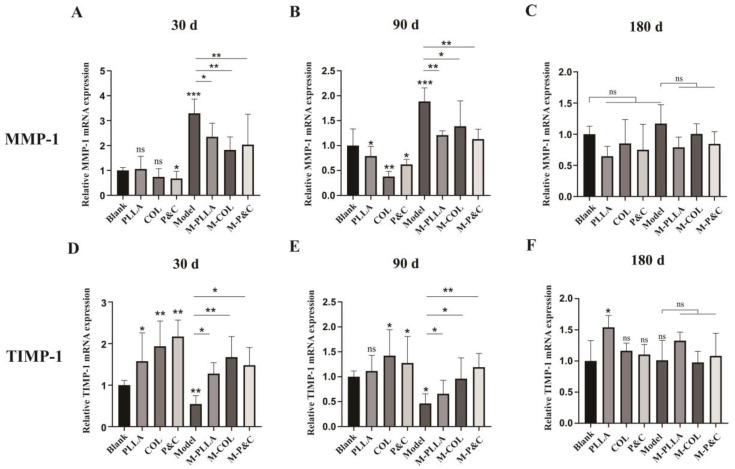
The mRNA expression levels of MMP-1 and TIMP-1 in rat skin tissues at different time points post-treatment. (**A**–**C**) MMP-1 mRNA expression. RT-qPCR analysis of MMP-1 transcript levels normalised to GAPDH after 30/90/180 days of injection. (**D**–**F**) TIMP-1 mRNA expression. RT-qPCR analysis of TIMP-1 transcript levels normalised to GAPDH after 30/90/180 days of injection. (*n* = 6; * *p* < 0.05; ** *p* < 0.01; *** *p* < 0.001; ‘ns’: *p* > 0.05).

Matrix metalloproteinase-1 (MMP-1) serves as a pivotal enzyme in the degradation of type I and III collagens. Its overexpression triggers the degradation of extracellular matrix components, thereby disrupting the structural integrity of collagen fibres in dermal tissues. This pathological process is clinically manifested as cutaneous ageing phenomena, including wrinkle formation and tissue depressions [37,38]. The tissue inhibitor of metalloproteinases 1 (TIMP-1) functions as a specific suppressor of matrix metalloproteinases, primarily targeting the enzymatic activity of MMP-1. Using real-time PCR, we quantitatively assessed the expression profiles of MMP1 and TIMP1 in skin tissue samples to characterise molecular alterations in collagen-degradation-related gene expression. The dynamic equilibrium between TIMPs and MMPs is critical for the coordinated regulation of collagen catabolism and anabolism [39]. The results are shown in Figure 7A–C. The results demonstrate that, 30 days post-injection, the MMP1 mRNA expression level in the model group was significantly elevated compared to the blank group. This upregulation indicates the onset of cutaneous senescence and enhanced collagen degradation in the rat skin tissues of the model group. Notably, the level of MMP1 expression in the rats treated with filler materials was significantly lower than that in the control group, suggesting that intervention with filler materials effectively inhibited MMP1 activity. In the D-galactose-treated rat group, the reduction in MMP1 gene expression may be attributed to the concurrent increase in TIMP1 expression within the skin tissue. As illustrated in Figure 7D–F, the results revealed a consistent inverse relationship, with the upregulation of TIMP1 gene expression aligning with the downregulation of MMP1 expression.

### 3.7. Epidermal Layer Thickness

**Figure 8:**
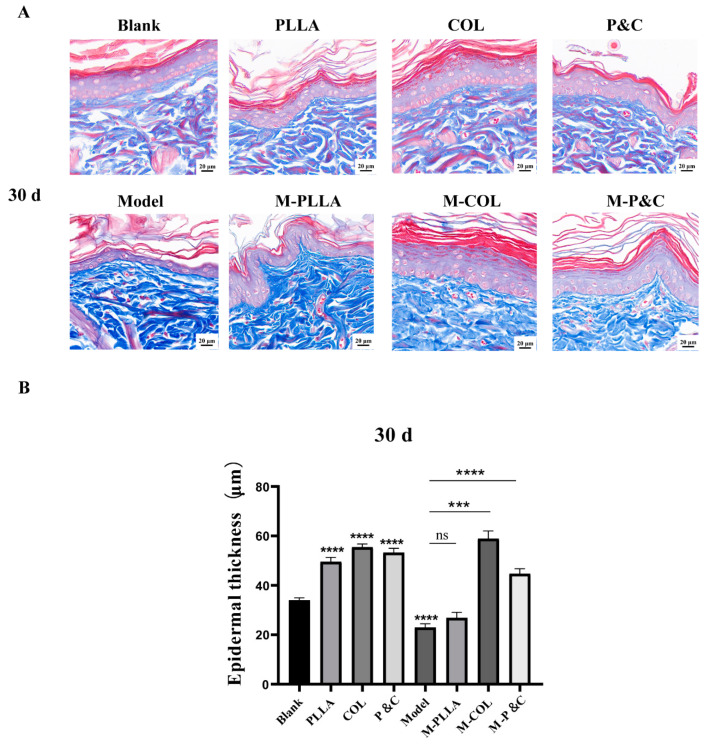
Masson’s trichrome staining and epidermal thickness analysis in rat skin tissues 30 days post-injection. (**A**) Representative histology images. Cross-sectional Masson’s trichrome-stained skin sections from each treatment group. Collagen fibres (blue), muscle fibres (red), and epidermal keratinocytes (purple) are differentially stained. Scale bar: 20 μm. (**B**) Epidermal thickness quantification. Distance from the stratum basale to the stratum corneum measured at five random sites per section using ImageJ. (*n* = 6; *** *p* < 0.001; **** *p* < 0.0001; ‘ns’: *p* > 0.05).

Masson staining was used to assess epidermal thickness changes across rat groups, with the results shown in Figure 8. At 30 days post-injection, the model group exhibited significantly reduced epidermal thickness compared to the blank control group, indicating that D-galactose induced damage to the rat skin tissue. In the M-PLLA group, epidermal thickness did not differ significantly from the model group. By contrast, both the M-COL and M-P&C groups showed substantial increases in epidermal thickness (*p* < 0.001), alongside denser basal cell arrangement and enhanced cellularity within the basal layer. It is hypothesised that, at 30 days post-injection, DuCol ameliorates epidermal atrophy in the ageing skin tissue and promotes significant accumulation of the epidermal layer matrix, thereby providing a robust filling effect. At 90 days post-injection, epidermal thickness in the M-PLLA, M-COL, and M-P&C groups was significantly greater than in the model group; however, the increase in the M-COL group was less pronounced than in the M-PLLA group (Figure S5). These findings indicate that PLLA begins to exert its filling and anti-wrinkle effects by 90 days post-injection, while DuCol maintains its anti-ageing activity. By 180 days post-injection, epidermal thickness did not differ between the model and blank control groups, likely reflecting normalisation of the acute ageing phenotype in rats following 180 days of recovery (Figure S6). The M-PLLA group showed no significant difference in epidermal thickness compared to the model group, whereas the M-COL group retained greater epidermal thickness than the model group. This suggests that the effect of DuCol sustains its early-stage effect on enhancing epidermal thickness, whereas PLLA does not demonstrate long-term maintenance of this parameter. Notably, epidermal thickness across all groups decreased at 180 days compared to 30 days and 90 days post-injection. This is hypothesised to reflect the natural onset of senescence in rats by 180 days post-injection, manifesting as age-related morphological changes.

### 3.8. Dermal Layer Thickness

**Figure 9:**
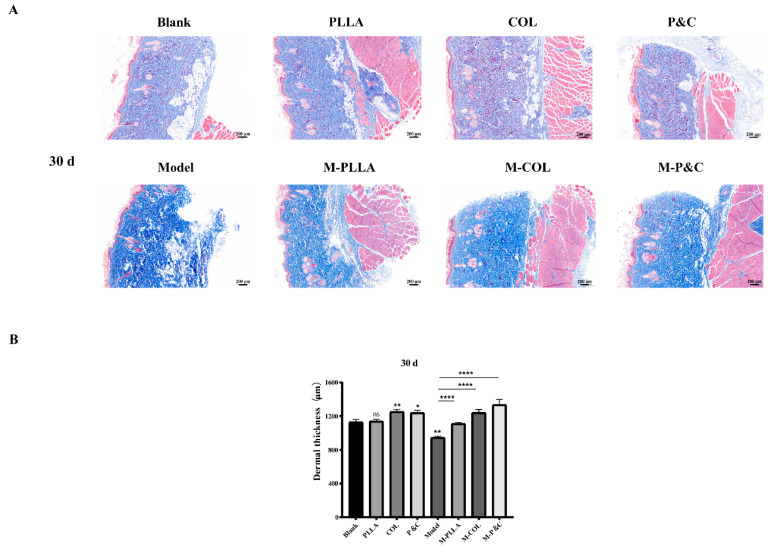
Masson’s trichrome staining and dermal thickness analysis in rat skin tissues 30 days post-injection. (**A**) Representative histology images. Cross-sectional Masson’s trichrome-stained skin sections from each treatment group. Collagen fibres (blue), muscle fibres (red), and epidermal keratinocytes (purple) are differentially stained. Scale bar: 200 μm. (**B**) Dermal thickness quantification. Distance from the stratum basale to the stratum corneum measured at 5 random sites per section using ImageJ. (*n* = 6; * *p* < 0.05; ** *p* < 0.01; **** *p* < 0.0001; ‘ns’: *p* > 0.05).

Masson staining was used to examine collagen fibre architecture in the rat skin following injection. As shown in Figure 9, at 30 days post-injection, dermal thickness in the COL and P&C groups increased significantly compared to the blank control group, with a more pronounced effect than in the M-PLLA group. This confirms that DuCol enhances early-stage collagen regeneration and fibre formation, underpinning its anti-ageing efficacy. By 90 days post-injection, the dermis in the PLLA, COL, and P&C groups exhibited densely packed collagen fibres, with significantly greater dermal thickness than the blank control (Figure S7). When compared to the model group, both M-COL and M-P&C groups showed greater dermal thickness than M-PLLA, indicating that DuCol sustains its capacity to promote dermal collagen fibre synthesis for up to 90 days. These findings indicate that the ability of DuCol to promote collagen fibre synthesis in the dermis can be maintained 90 days after injection. At 180 days post-injection, all treatment groups demonstrated stable dermal thickness without reduction (Figure S8). These findings indicate that DuCol, PLLA, and their combination (P&C) enhance rat dermal thickness, with the combined formulation exhibiting superior anti-ageing effects.

## 4. Discussion

With advancing age, collagen content and metabolic rate in human skin decline, accompanied by structural alterations in collagen fibres. These changes induce intra-dermal collagen fibre cross-linking and stiffening, reduced intercellular mucopolysaccharide content, elastic fibre rupture, and lipid atrophy. Collectively, these processes give rise to a cascade of ageing manifestations, including loss of elasticity in the skin, cutaneous senescence and dyschromia, and the appearance of wrinkles and pigmentation [40]. The implantation of medical devices is currently the main strategy for repairing tissue defects. This in vivo tissue engineering strategy exhibits substantial promise in tissue regeneration offering significant future benefits for patients. However, it imposes stringent requirements on implant materials [1]. In skin tissue engineering, a variety of materials, including natural macromolecules, such as collagen and HA, and synthetic polymers, such as polyacrylamide hydrogel (PAAG) and polymethyl methacrylate (PMMA), have been studied for their potential applications. The ideal facial tissue filler material should be safe, effective, and biocompatible. The high hydrophilicity of hyaluronic acid influences the mechanical properties of deep adipose tissue at the injection site, often inducing hardness in subcutaneous fat. Its short half-life (1–2 days) results in rapid degradation and a transient therapeutic effect. While chemical cross-linking can prolong the efficacy of hyaluronic acid injections, this process compromises the purity of the injectate and elevates the risk of adverse reactions, including skin erythema, acneiform eruptions, and haematoma [41]. Synthetic materials, such as polylactic acid (PLA) and polyglycolic acid (PGA), while non-biodegradable in vivo and suitable for permanent injectable filling, pose challenges. Under sustained external forces, including extrusion and friction, their microparticles migrate within tissues, leading to a high incidence of granulomas. Furthermore, once complications arise, these materials cannot be safely removed, often triggering immune responses, such as local inflammation and ischaemia [42,43]. By contrast, recombinant humanised collagen, a biosynthetic material with a customisable amino acid sequence and promising biological functionality, mitigates the risks of viral transmission and immune hypersensitivity [1]. Building on the characterisation of DuCol, this study utilises a skin ageing model to evaluate its biological effects in vivo.

Our findings demonstrate that DuCol exhibits early-phase therapeutic efficacy in a D-galactose-induced metabolic ageing model, complementing and mechanistically expanding upon the work of Wang et al. [1] on recombinant humanised type III collagen (rhCol III) in UV-photoaged skin. While both studies employed collagen-based interventions in animal models, the distinct ageing induction methods—D-galactose (mimicking metabolic ageing) versus UV radiation (inducing photoageing)—produced divergent pathophysiological responses. Wang et al. reported rhCol III-mediated mitigation of UV-induced epidermal thickening and elastin disorganisation through MMP-3 suppression and oxidative stress neutralisation, whereas our model revealed DuCol’s capacity to reverse D-galactose-driven dermal thinning with expression patterns of TGFβ-1 and Smad3 suggesting involvement of the TGF-β/Smad3 pathway. While the ageing triggers differ, these studies collectively demonstrate the versatility of recombinant collagens in addressing distinct ageing etiologies. DuCol’s early-phase TGF-β/Smad3-dependent collagen induction in metabolic ageing complements rhCol III’s MMP-3-suppressive effects in photo ageing, highlighting the importance of collagen type and structural design in tailoring anti-ageing strategies. The divergent models reveal context-dependent mechanisms: metabolic ageing requires stimulation of collagen biosynthesis (DuCol’s strength), whereas photoageing demands inhibition of matrix degradation (rhCol III’s focus). This underscores the need for a model-specific material design in translational research. Structural distinctions critically influenced functional outcomes. Wang et al. utilised a homotrimeric type III collagen fragment that enhanced dermal repair via α2β1 integrin-mediated fibroblast interactions, whereas our chimeric DuCol combining type I (Gly1076-Pro1202) and III (Gly594-Pro846) domains with hybrid integrin-binding motifs (GPAGEK, GAPGER) exhibited superior early-stage bioactivity, stimulating fibroblast proliferation and adhesion within 24 h. Temporal efficacy profiles further differentiated these collagens: rhCol III induced progressive collagen deposition over 8 weeks, while DuCol achieved rapid COL1A1 induction (peak at 30 days) followed by biodegradation-dependent efficacy decline, contrasting with PLLA delayed but sustained effects. Our combinatorial PLLA/DuCol strategy bridged these temporal limitations, synergizing early-phase matrix synthesis with prolonged structural support. Mechanistically, the observed upregulation of the TGF-β/Smad3 pathway components and concomitant increase in COL1A1 expression suggest that DuCol may enhance collagen biosynthesis, potentially through TGF-β/Smad3 signalling activation. This positions recombinant collagens as complementary tools tailored to distinct ageing etiologies—rhCol III counteracting oxidative stress, and DuCol addressing collagen depletion via pathway modulation.

Notably, while native collagen I has been widely used in dermal regeneration for its structural and bioactive properties, DuCol offers distinct advantages and limitations. Compared to animal-derived collagen I (e.g., bovine or porcine), DuCol’s recombinant humanised design eliminates the risks of xenogeneic immunogenicity and pathogen transmission, while maintaining key functional domains (e.g., integrin-binding motifs) necessary for fibroblast activation. However, unlike full-length native collagen I (300 kDa) that forms stable triple-helical fibrils, DuCol’s engineered fragment lacks the complete helical structure, which may explain its faster biodegradation and transient bioactivity. Despite this, DuCol demonstrated superior early-phase collagen I deposition compared to PLLA, suggesting that its truncated structure retains sufficient signalling capacity to stimulate endogenous collagen synthesis, bypassing the need for slow enzymatic remodelling of bulk animal-derived collagen. This balance between rapid bioactivity and controlled degradation positions DuCol as a complementary material to native collagen I in applications requiring immediate matrix reinforcement.

The development of recombinant collagens has expanded the toolkit for biomedical applications, yet most synthetic collagens prioritise either structural mimicry or simplified production, often compromising functionality. DuCol distinguishes itself through three key innovations, including hybrid domain design, balanced bioactivity–degradation profile, and humanised safety and scalability. Hybrid domain design: Unlike homotypic recombinant collagens (e.g., rhCol III or yeast-derived collagen-like peptides), DuCol integrates human type I and III collagen domains (Gly1076–Pro1202 of type I and Gly594–Pro846 of type III) into a single chimeric molecule. This design preserves integrin-binding motifs (e.g., GFOGER from type I, GAPGER from type III) while eliminating non-functional regions, enabling simultaneous engagement of multiple fibroblast receptors (e.g., α2β1 and DDR2) to amplify collagen synthesis. Balanced bioactivity–degradation profile: Many synthetic collagens exhibit either rapid degradation (e.g., short collagen peptides) or non-physiological persistence (e.g., PEGylated collagens). DuCol’s engineered molecular weight (28 kDa) and filamentous porous structure achieve intermediate biodegradation kinetics—sufficiently stable to sustain TGF-β/Smad3 signalling for 30–90 days, yet fully resorbable by 180 days. This contrasts with PLLA’s indefinite persistence and aligns with clinical needs for transient yet impactful dermal remodelling. Humanised safety and scalability: While bacterial expression systems (e.g., *E. coli*) are commonly used for recombinant collagen production, codon optimisation and soluble expression strategies enabled high-yield DuCol synthesis without mammalian post-translational modifications. This ensures batch-to-batch consistency and eliminates zoonotic risks—a critical advantage over animal-derived collagens and some plant-expressed variants requiring complex glycosylation. These features position DuCol as a versatile and clinically translatable biomaterial that bridges the gap between transient bioactive peptides and permanent synthetic fillers.

The absence of the characteristic 220 nm positive peak in DuCol’s CD spectrum (Figure S1D) initially appears inconsistent with its predicted triple-helical architecture (Figure 1A). This discrepancy, however, likely stems from the technical limitations and structural dynamics inherent to engineered collagen fragments rather than a complete absence of helical organisation. In parallel experiments under identical conditions, native bovine collagen exhibited similarly weak 220 nm signals, suggesting that instrumental sensitivity thresholds or low sample concentrations may obscure subtle helical signatures. Computational modelling indicates DuCol’s truncated sequence (28 kDa) retains the capacity for triple-helix formation, albeit with reduced structural rigidity compared to full-length collagen (300 kDa), potentially resulting in a dynamic equilibrium between folded and partially unfolded states. Such flexibility could attenuate the 220 nm peak while preserving sufficient helical content to mediate bioactivity, as evidenced by robust fibroblast proliferation, adhesion, and COL1A1 upregulation (Figure 2 and Figure 3). To resolve this ambiguity, future work will employ synchrotron radiation CD (enhanced sensitivity) and thermal denaturation assays to quantify helical stability. Nevertheless, the retained 198 nm negative peak and compelling functional data collectively affirm that DuCol adopts a collagen-like secondary structure critical to its therapeutic efficacy, reconciling predictive modelling with empirical observations.

## 5. Conclusions

The use of recombinant humanised collagen circumvents the limitations of animal-derived collagens, establishing it as an ideal substrate for surgical implants and tissue-engineered medical products. In this study, a D-galactose-induced rat ageing model was employed to evaluate recombinant humanised collagen as a facial filler material. Its effectiveness and safety in retarding skin ageing were evaluated across protein, tissue structural, and gene expression levels at multiple time points. PLLA was included as a comparator implant due to its established efficacy, with the dual objectives of benchmarking performance and investigating whether material combination could enhance clinical outcomes. The results revealed that DuCol mitigates skin ageing by exerting early-phase effects. Unlike PLLA, which initiates activity after 90 days, DuCol elicited measurable improvements as early as 30 days post-injection, with sustained benefits for up to 90 days. This temporal profile indicates that recombinant humanised collagen is particularly suited to patients seeking rapid facial rejuvenation and short-term improvement of skin ageing, whereas PLLA remains the preferred choice for individuals requiring filler effects lasting three months or longer. In conclusion, this research underscores the translational potential of recombinant humanised collagen in medical cosmetology, providing a robust foundation for its clinical implementation and further mechanistic investigation.

## Figures and Tables

**Table 1:** The main information of the experimental animals and implants.

Groups	Number	D-Gal Injection	Implants (Concentration; Volume)
Blank	12	−	Sodium carboxymethyl cellulose solution (1%; 200 μL)
PLLA	12	−	PLLA in 1% sodium carboxymethyl cellulose solution (68 mg/mL; 200 μL)
COL	12	−	DuCol in 1% sodium carboxymethyl cellulose solution (4 mg/mL; 200 μL)
P&C	12	−	PLLA and DuCol in 1% sodium carboxymethyl cellulose solution (68 mg/mL, 4 mg/mL; 200 μL)
Model	12	+	Sodium carboxymethyl cellulose solution (1%; 200 μL)
M-PLLA	12	+	PLLA in 1% sodium carboxymethyl cellulose solution (68 mg/mL; 200 μL)
M-COL	12	+	DuCol in 1% sodium carboxymethyl cellulose solution (4 mg/mL; 200 μL)
M-P&C	12	+	PLLA and DuCol in 1% sodium carboxymethyl cellulose solution (68 mg/mL, 4 mg/mL; 200 μL)

**Table 2:** The primer sequences of the target gene for RT-qPCR.

Gene Name	Direction	Sequence (5′–3′)
*GAPDH*	Forward	CTCTCTGCTCCTCCCTGTT
	Reverse	TACGGCCAAATCCGTTCAC
*COL1A1*	Forward	GACTGGAAGAGCGGAGAGTA
	Reverse	TGGGCTGATGTACCAGTTCT
*TGFβ-1*	Forward	AGCAACAATTCCTGGCGTTA
	Reverse	AGCCCTGTATTCCGTCTCC
*Smad3*	Forward	AGGAGAAGTGGTGCGAGAA
	Reverse	CATCCAGTGACCTGGGGAT
*TIMP1*	Forward	AGAGACACGCTAGAGCAGATAC
	Reverse	CCAGGTCCGAGTTGCAGAA
*MMP1*	Forward	TCCAGGCTTTATATGGGCCTT
	Reverse	TCACCTCTCCCCTAAACGTAG

**Table 3:** The amino acid sequence of DuCol.

Collagen	Sequence
DuCol	MGPQGIAGQRGVVGLPGQRGERGFPGLPGPSGEPGKQGPSGAS GERGPPGPMGPPGLAGPPGESGREGAPGAEGSPGRDGSPGAKGD RGETGPAGPPGAPGAPGAPGPVGPAGKSGDRGETGPAGPAGERG GPGGPGPQGPPGKNGETGPQGPPGPTGPGGDKGDTGPPGPQGLQ GLPGTGGPPGENGKPGEPGPKGDAGAPGAPGGKGDAGAPGERG PPGLAGAPGLRGGAGPPGPEGGKGAAGPPGPPGAAGTPGLQGM PGPPGPCCGGG

## Data Availability

The original contributions presented in the study are included in the article/Supplementary Material, further inquiries can be directed to the corresponding author.

## Supplementary Materials

The following supporting information can be downloaded at: https://www.mdpi.com/article/10.3390/bioengineering12050510/s1, Figure S1: Characterization of DuCol; Figure S2: SOD enzyme activity units and MDA levels in rat plasma; Figure S3: Sirius red staining and collagen content analysis in rat skin tissues 90 days post-injection; Figure S4: Sirius red staining and collagen content analysis in rat skin tissues 180 days post-injection; Figure S5: Masson’s trichrome staining and epidermal thickness analysis in rat skin tissues 90 days post-injection; Figure S6: Masson’s trichrome staining and epidermal thickness analysis in rat skin tissues 180 days post-injection; Figure S7: Masson’s trichrome staining and dermal thickness analysis in rat skin tissues 90 days post-injection; Figure S8: Masson’s trichrome staining and dermal thickness analysis in rat skin tissues 180 days post-injection.
