# Supplementary material for: In Vivo Evaluation of the Anti-Skin-Ageing Bioactivity of a Recombinant Dual Humanised Collagen and Poly-L-Lactic Acid
Source: Bioengineering (Basel). 2025 May 12;12(5):510. doi: 10.3390/bioengineering12050510 (PMC12109386; doi:10.3390/bioengineering12050510)
Supplement: Supplementary file 1 [file bioengineering-12-00510-s001.zip › Supplementary Material.pdf]

Supplementary Data

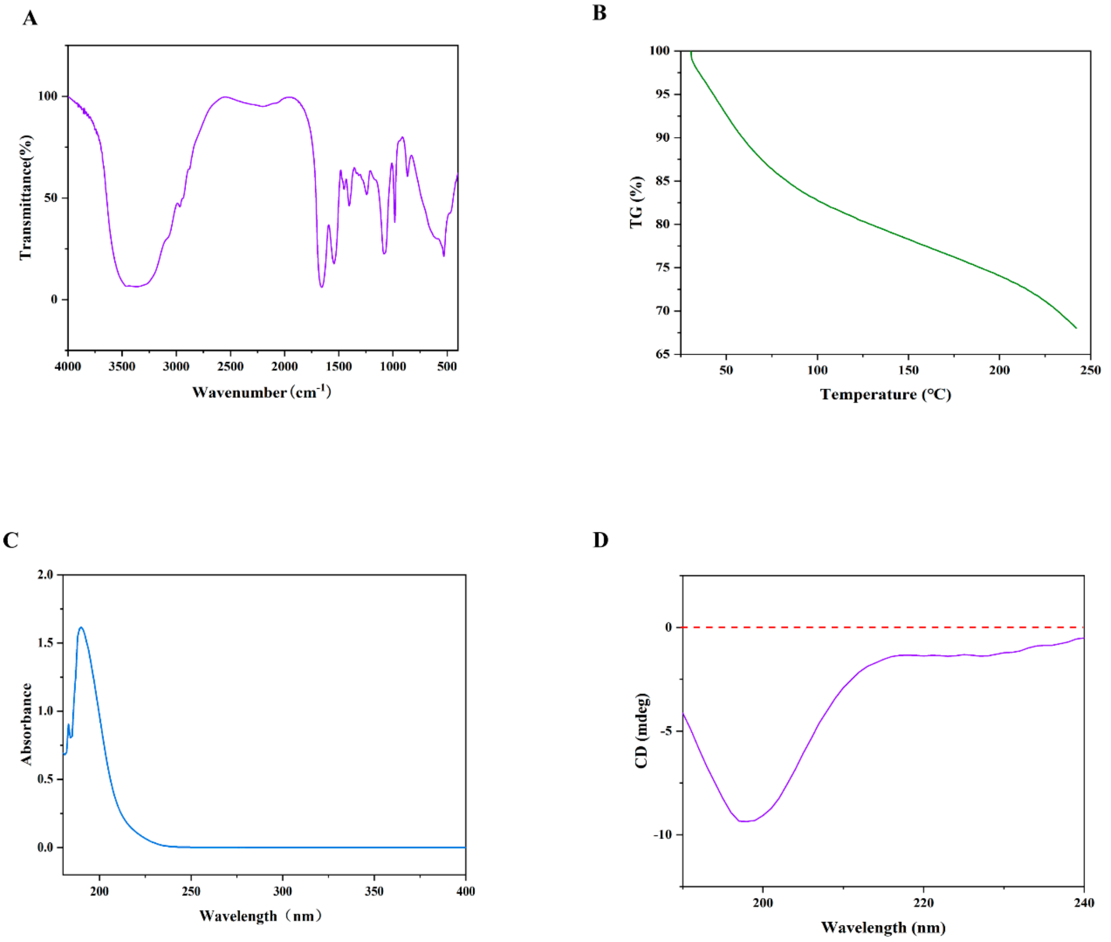

Fig.S1 Characterization of DuCol. A. Infrared spectrogram of DuCol. B. TGA of DuCol. C. UV spectrogram of DuCol. D.CD of DuCol.

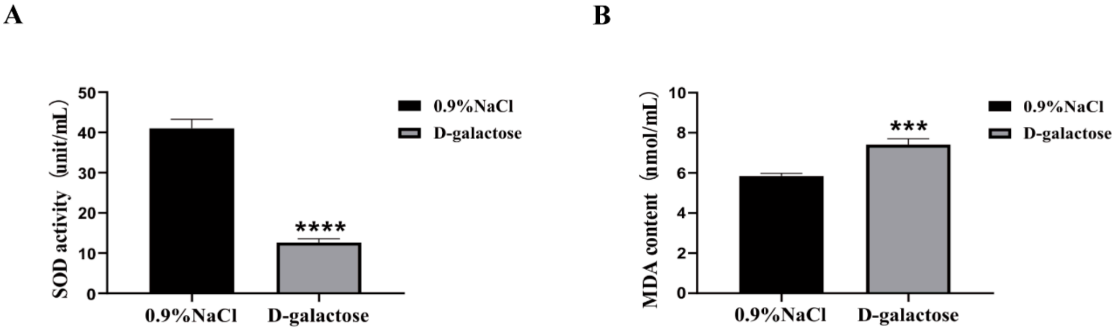

Fig.S2 SOD enzyme activity units and MDA levels in rat plasma. A) SOD enzyme activity unit in normal and aging rats; B) MDA content in normal and aging rats.

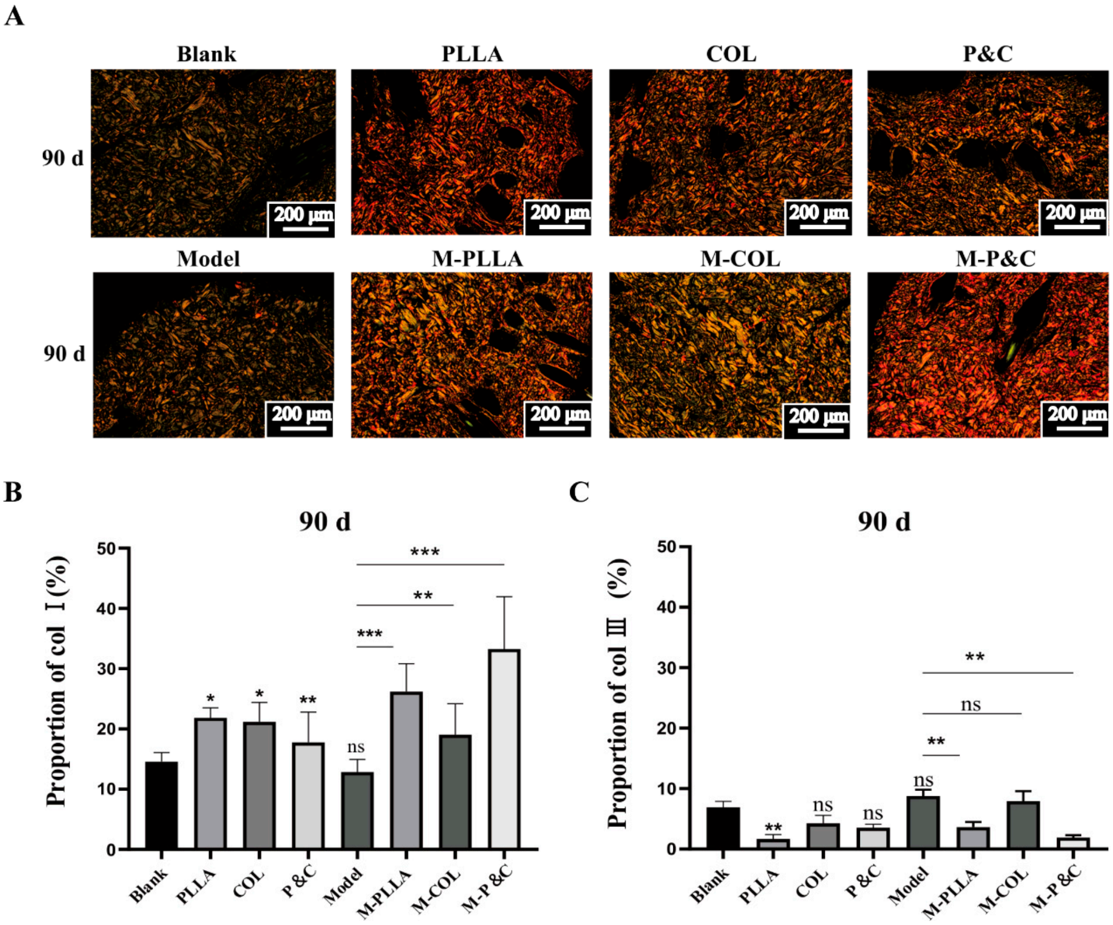

Fig. S3 Sirius red staining and collagen content analysis in rat skin tissues 90 days post-injection. A) Representative polarized light microscopy images. Sirius red-stained sections of rat dermis from each treatment group. Type I collagen fibers (red/yellow birefringence) and type III collagen fibers (green birefringence) were differentiated under polarized light microscope. Scale bar: 200 μm. B) Quantification of type I collagen. Percentage of type I collagen area relative to total dermal area, analyzed using ImageJ. C) Quantification of type III collagen. Percentage of type III collagen area relative to total dermal area. (n=6; *P < 0.05; **P < 0.01; ***P < 0.001; ****P < 0.0001; 'ns': P > 0.05).

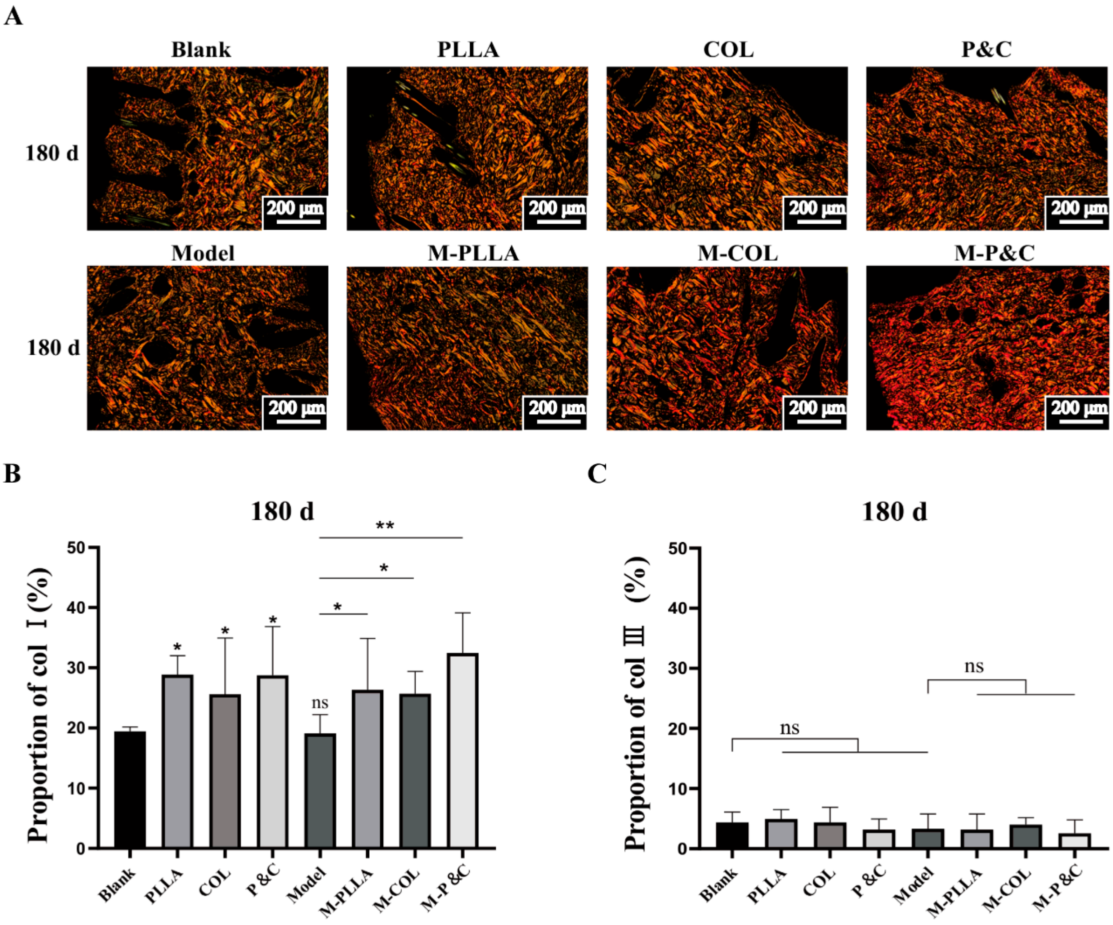

Fig. S4 Sirius red staining and collagen content analysis in rat skin tissues 180 days post-injection. A) Representative polarized light microscopy images. Sirius red-stained sections of rat dermis from each treatment group. Type I collagen fibers (red/yellow birefringence) and type III collagen fibers (green birefringence) were differentiated under polarized light microscope. Scale bar: 200 μm. B) Quantification of type I collagen. Percentage of type I collagen area relative to total dermal area, analyzed using ImageJ. C) Quantification of type III collagen. Percentage of type III collagen area relative to total dermal area. (n=6; *P < 0.05; **P < 0.01; ***P < 0.001; ****P < 0.0001; 'ns': P > 0.05).

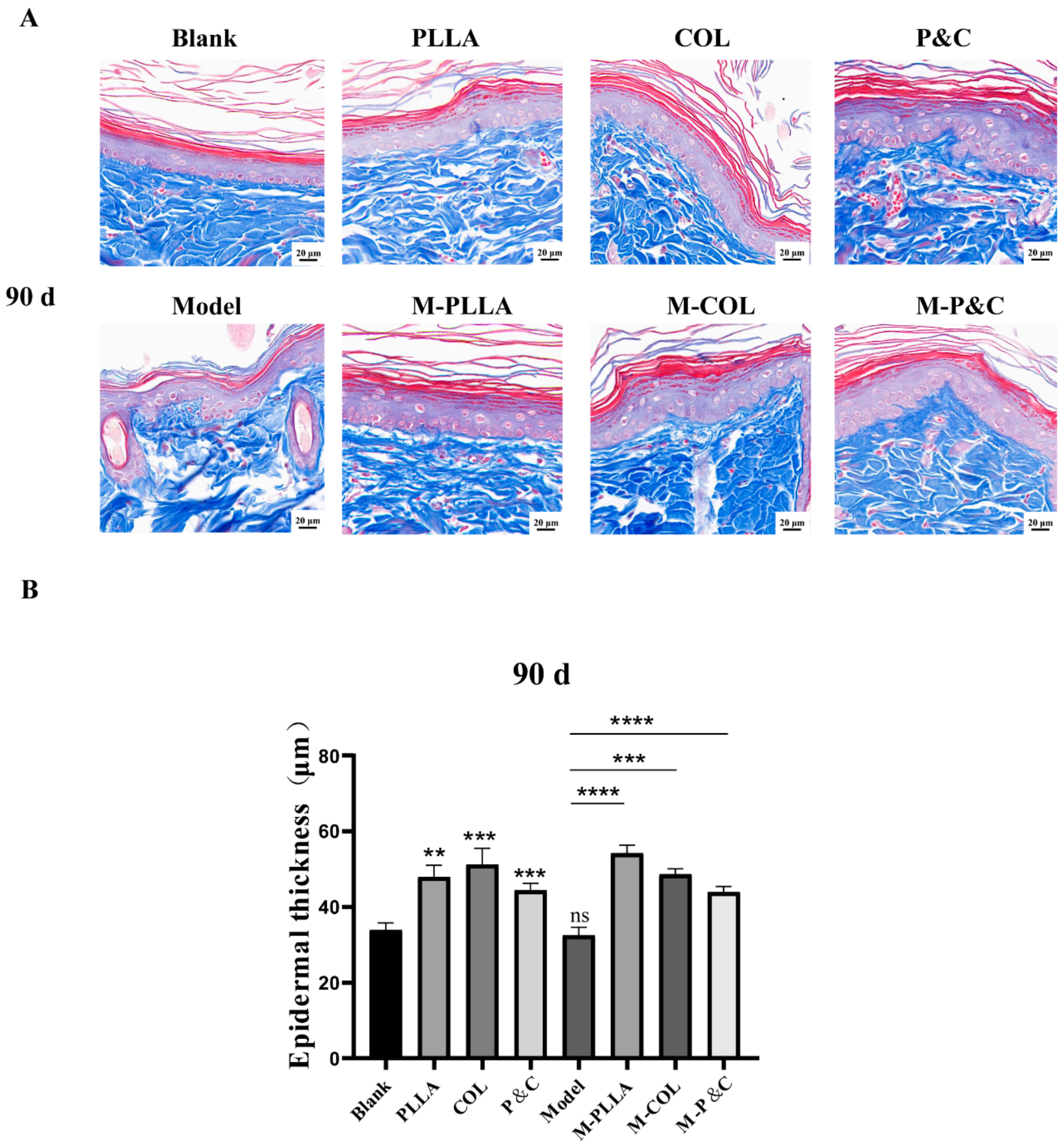

Fig. S5 Masson's trichrome staining and epidermal thickness analysis in rat skin tissues 90 days post-injection. A) Representative histology images. Cross-sectional Masson's trichrome-stained skin sections from each treatment group. Collagen fibers (blue), muscle fibers (red), and epidermal keratinocytes (purple) are differentially stained. Scale bar: 20 μm. B) Epidermal thickness quantification. Distance from the stratum basale to the stratum corneum measured at 5 random sites per section using Image J. (n=6; *$P < 0.05$; **$P < 0.01$; ***$P < 0.001$; ****$P < 0.0001$; 'ns': $P > 0.05$).

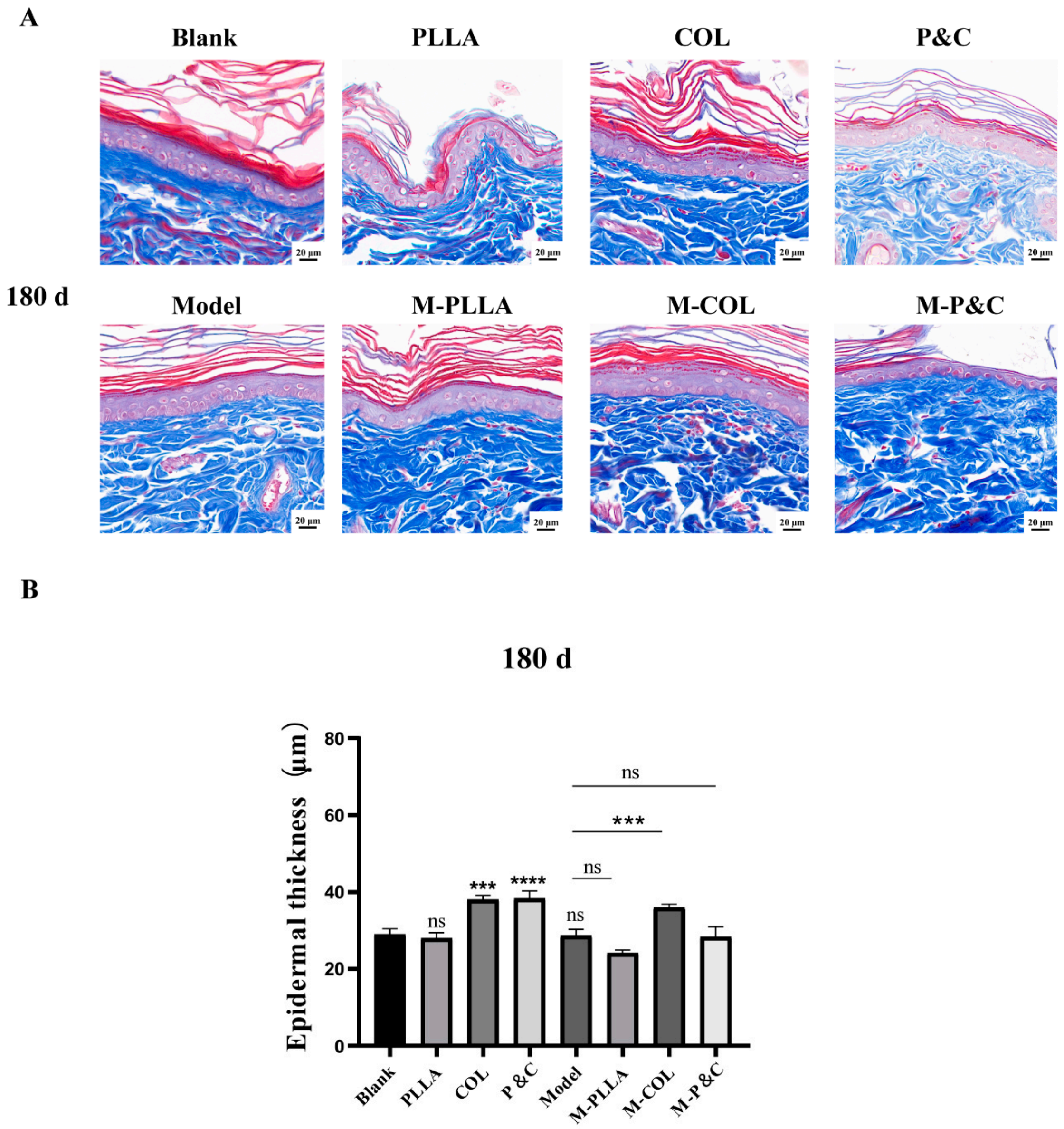

Fig. S6 Masson's trichrome staining and epidermal thickness analysis in rat skin tissues 180 days post-injection. A) Representative histology images. Cross-sectional Masson's trichrome-stained skin sections from each treatment group. Collagen fibers (blue), muscle fibers (red), and epidermal keratinocytes (purple) are differentially stained. Scale bar: 20 μm. B) Epidermal thickness quantification. Distance from the stratum basale to the stratum corneum measured at 5 random sites per section using Image J. (n=6; *P < 0.05; **P < 0.01; ***P < 0.001; ****P < 0.0001; 'ns': P > 0.05).

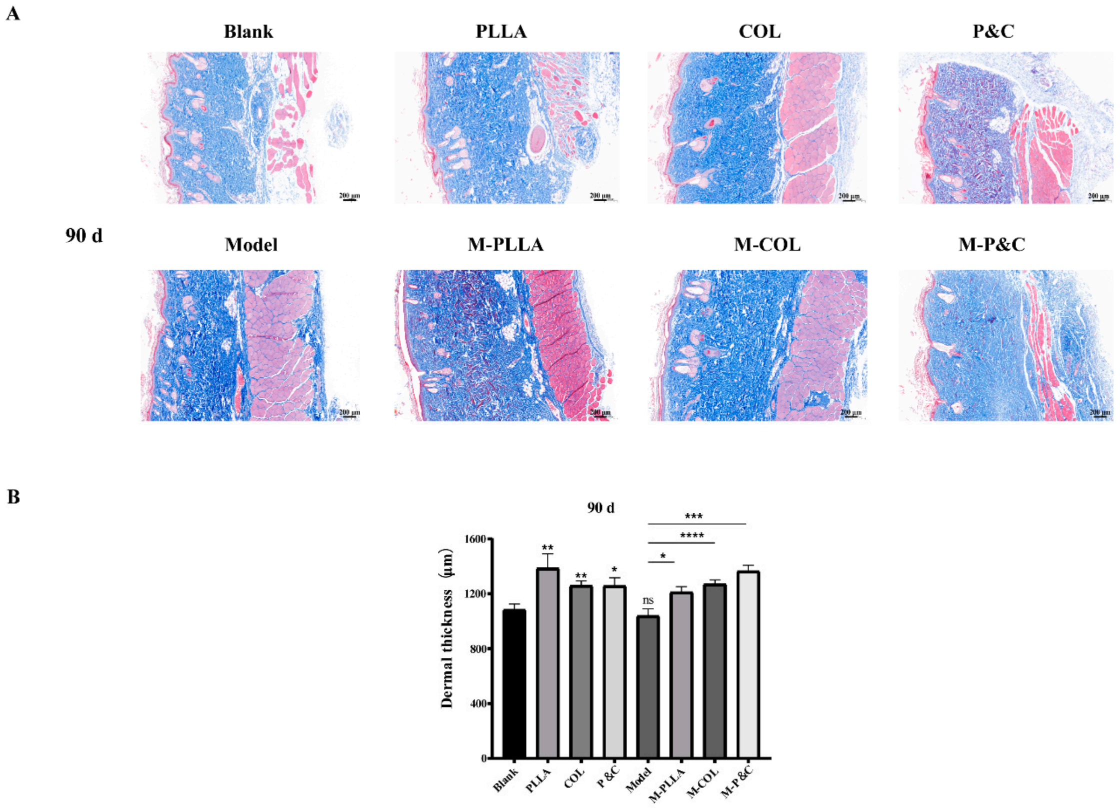

Fig. S7 Masson's trichrome staining and dermal thickness analysis in rat skin tissues 90 days post-injection. A) Representative histology images. Cross-sectional Masson's trichrome-stained skin sections from each treatment group. Collagen fibers (blue), muscle fibers (red), and epidermal keratinocytes (purple) are differentially stained. Scale bar: 200 μm. B) Dermal thickness quantification. Distance from the stratum basale to the stratum corneum measured at 5 random sites per section using Image J.

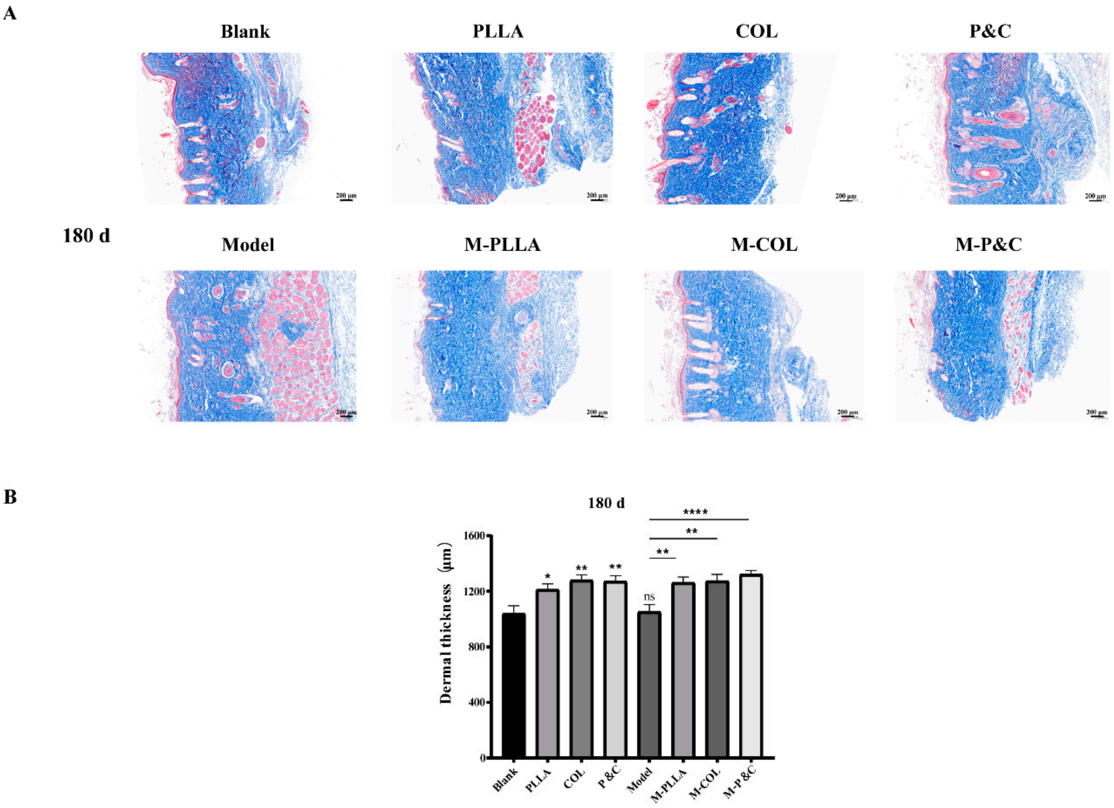

Fig. S8 Masson's trichrome staining and dermal thickness analysis in rat skin tissues 180 days post-injection. A) Representative histology images. Cross-sectional Masson's trichrome-stained skin sections from each treatment group. Collagen fibers (blue), muscle fibers (red), and epidermal keratinocytes (purple) are differentially stained. Scale bar: 200 μm. B) Dermal thickness quantification. Distance from the stratum basale to the stratum corneum measured at 5 random sites per section using Image J.
