# Supplementary figures and images for: In Vivo Evaluation of the Anti-Skin-Ageing Bioactivity of a Recombinant Dual Humanised Collagen and Poly-L-Lactic Acid
Source: Bioengineering (Basel). 2025 May 12;12(5):510. doi: 10.3390/bioengineering12050510 (PMC12109386; doi:10.3390/bioengineering12050510)

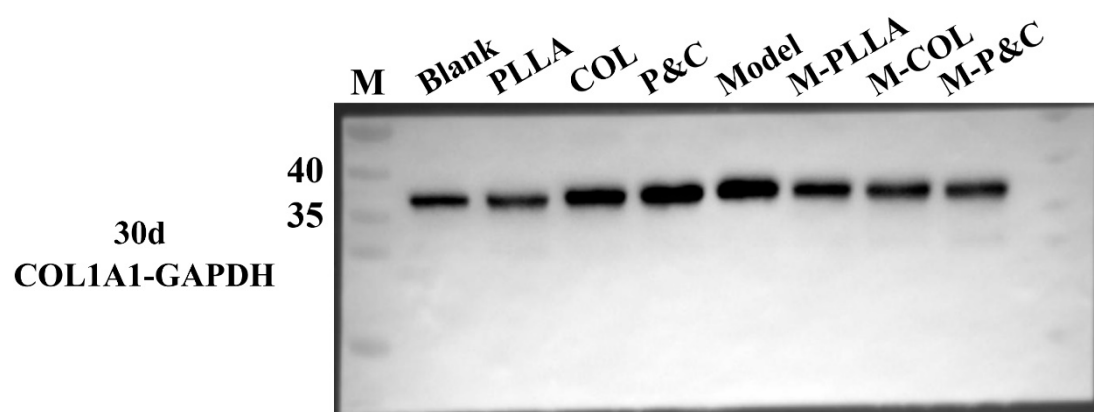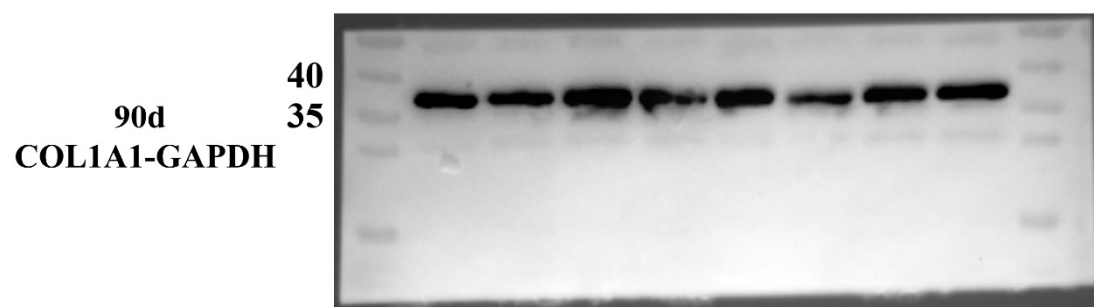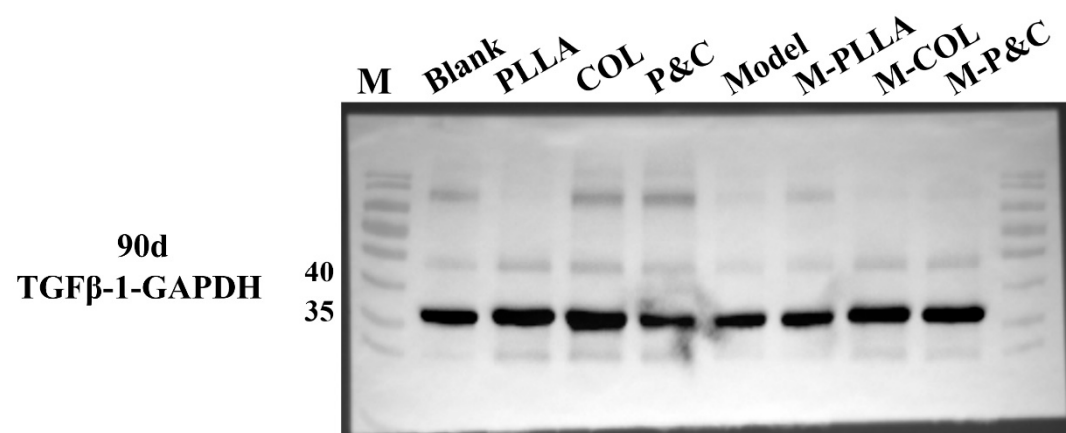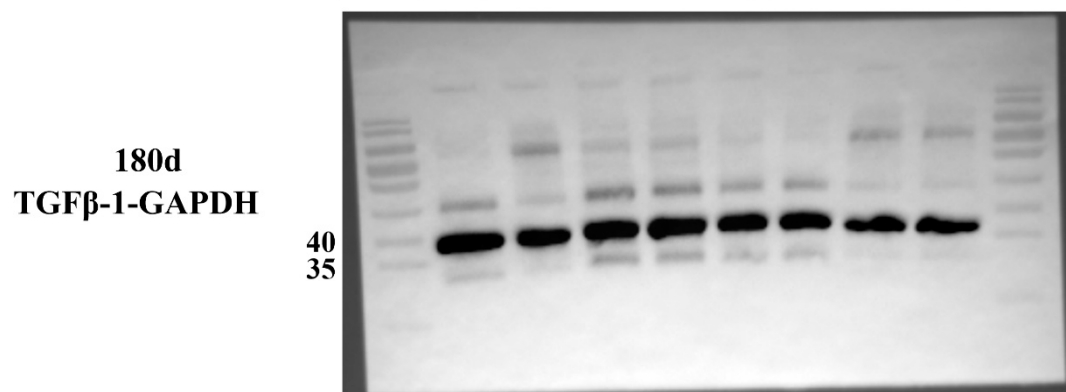

Supplement: Supplementary file 1 [file bioengineering-12-00510-s001.zip › Supplementary GAPDH blot original image.pdf]
